# Erectile Dysfunction Is a Hallmark of Cardiovascular Disease: Unavoidable Matter of Fact or Opportunity to Improve Men’s Health?

**DOI:** 10.3390/jcm10102221

**Published:** 2021-05-20

**Authors:** Dimitri Yannas, Francesca Frizza, Linda Vignozzi, Giovanni Corona, Mario Maggi, Giulia Rastrelli

**Affiliations:** 1 Department of Experimental and Clinical Biomedical Sciences “Mario Serio”, University of Florence, 50121-50145 Florence, Italy; dimitriyannas@gmail.com (D.Y.); linda.vignozzi@unifi.it (L.V.); mario.maggi@unifi.it (M.M.); 2 Andrology, Women’s Endocrinology and Gender Incongruence Unit, Careggi Teaching Hospital, 50121-50145 Florence, Italy; 3 Endocrinology Unit, Medical Department, Azienda Usl Maggiore-Bellaria Hospital, 40121-40141 Bologna, Italy; francyfrizza@gmail.com (F.F.); jocorona@libero.it (G.C.); 4 Endocrinology Unit, Careggi Teaching Hospital, 50121-50145 Florence, Italy

**Keywords:** erectile dysfunction, cardiovascular risk factors, unconventional risk factors, major adverse cardiovascular events

## Abstract

Erectile dysfunction (ED) is an early manifestation of cardiovascular (CV) disease. For this reason, men with ED should be carefully assessed for CV risk factors in order to prevent future major adverse CV events (MACE). Traditional risk factors are not found in all subjects at high CV risk. In fact, a relevant proportion of MACE occurs in men who are apparently risk factor free. In men with ED, it is important to take into account not only traditional risk factors but also unconventional ones. Several parameters that derive from good clinical assessment of subjects with ED have proven to be valuable predictors of MACE. These include family history of cardiometabolic events, alcohol abuse, fatherhood, decreased partner’s sexual interest, severe impairment in erection during intercourse or during masturbation, impaired fasting glucose, increased triglycerides, obesity even without metabolic complications, decreased penile blood flows or impaired response to an intra-cavernosal injection test. Recognizing these risk factors may help in identifying, among subjects with ED, those who merit stricter lifestyle or pharmacological interventions to minimize their CV risk. Effective correction of risk factors in ED men considered as high risk, besides reducing CV risk, is also able to improve erectile function.

## 1. Introduction

Non-communicable diseases are responsible for more than 70% of deaths worldwide [1] as they are largely the main mortality cause in high-income countries (88% of all causes of death). Globally, cardiovascular (CV) diseases (CVD) are the most frequent occurrence among the non-communicable diseases. In particular, ischemic heart disease (IHD) and stroke remain the leading causes of mortality, together causing more than 17.8 million deaths in 2017 [2]. Even more importantly, the increase of 17.3% and 12.1% in Years of Life Lost observed from 2007 to 2017 for IHD and stroke, respectively [2], denotes that CVD mortality is affecting younger bands of the population more and more. Besides mortality, disability consequent to a CV event, particularly among younger populations, is a further source of concern because it contributes heavily to the socio-economic burden caused by CVD.

The increasing incidence and adverse consequences of CVDs has driven research efforts to identify risk factors and, in particular, to recognize modifiable factors and the possible strategies to prevent or revert their occurrence. Lifestyle measures and specific medications are now largely used to correct these well-known risk factors. The widespread increasing focus on the achievement of desirable targets has shed light on CV risk in subjects without or with well-controlled risk factors. This is called residual risk and demonstrates that, besides conventional risk factors, other conditions modulate the individual risk of experiencing major adverse cardiovascular events (MACE). Research in several fields—more or less close to cardiology—has identified numerous lifestyle, clinical, biochemical or instrumental parameters that can predict MACE in general or in specific populations. The recognition of factors other than conventional parameters provides the opportunity to identify subjects that, despite being classified as at low risk according to conventional risk factors, deserve stricter follow-up and treatment measures.

Erectile dysfunction (ED) is a frequent condition worldwide [3] and its prevalence has been estimated to have more than doubled over the last 30 years [4]. The global ED prevalence varies from 13–71% [5]. This wide range is due to the different methods for the assessment of ED used by various studies but also to the different risk factor representation among the studied populations. Ageing is the first risk factor that has been incontrovertibly associated with ED [3]. However, other factors have further emerged as associated with ED. Interestingly, most of these are implicated in the occurrence of atherosclerosis and therefore are also known risk factors for MACE.

The overlap of CVD and ED risk factors explains the epidemiologic relationship between these conditions. ED is present in more than 50% of men with a history of CVD [6] and, on the other hand, one out of eight men with ED reports previous CVD [7]. This suggests that ED is a symptom that occurs frequently before a MACE. Accordingly, a recent meta-analysis of observational longitudinal studies has shown a 59% and 34% increased relative risk of IHD and stroke, respectively, in men with ED [8]. The temporal relationship between ED and MACE has earned ED the status of a “risk factor” for CVD. However, this term is not appropriate because ED is indeed on the temporal trajectory of CVD, but it is not on its pathogenic pathway. It has been nearly 20 years since Montorsi explained this association with his well-known “artery size hypothesis”. This argues that ED and CVD are different manifestations of a systemic arterial damage and, due to the smaller diameter of penile arteries, ED occurs earlier than IHD, stroke or peripheral artery disease (PAD) with an equal extent of artery damage [9]. Accordingly, it is estimated that ED precedes the first CV event by three years on average [10].

The aim of this narrative review is to summarize the available knowledge on the role of conventional and unconventional CV risk factors in ED men, either for sexual or CV outcomes. We also aim to summarize the available evidence on the amelioration of CV risk factors in ED subjects and their consequences for erectile outcomes.

## 2. Methods

A comprehensive search of the relevant literature in Medline was performed using the following terms: (“heart disease risk factors” (MeSH Terms) OR (“heart” (All Fields) AND “disease” (All Fields) AND “risk” (All Fields) AND “factors” (All Fields)) OR “heart disease risk factors” (All Fields) OR (“cardiovascular” (All Fields) AND “risk” (All Fields) AND “factors” (All Fields)) OR “cardiovascular risk factors” (All Fields)) AND (“erectile dysfunction” (MeSH Terms) OR (“erectile” (All Fields) AND “dysfunction” (All Fields)) OR “erectile dysfunction” (All Fields)).

For the section on CV risk modification, the following search terms were used: (“medic” (All Fields) OR “medical” (All Fields) OR “medicalization” (MeSH Terms) OR “medicalization” (All Fields) OR “medicalizations” (All Fields) OR “medicalize” (All Fields) OR “medicalized” (All Fields) OR “medicalizes” (All Fields) OR “medicalizing” (All Fields) OR “medically” (All Fields) OR “medicals” (All Fields) OR “medicated”(All Fields) OR “medications” (All Fields) OR “medics” (All Fields) OR “pharmaceutical preparations” (MeSH Terms) OR (“pharmaceutical” (All Fields) AND “preparations” (All Fields)) OR “pharmaceutical preparations” (All Fields) OR “medication” (All Fields) OR “medications” (All Fields)) AND (“heart disease risk factors” (MeSH Terms) OR (“heart” (All Fields) AND “disease” (All Fields) AND “risk” (All Fields) AND “factors” (All Fields)) OR “heart disease risk factors” (All Fields) OR (“cardiovascular” (All Fields) AND “risk” (All Fields) AND “factors” (All Fields)) OR “cardiovascular risk factors” (All Fields)) AND (“erectile dysfunction” (MeSH Terms) OR (“erectile” (All Fields) AND “dysfunction” (All Fields)) OR “erectile dysfunction” (All Fields)) and (“modification” (All Fields) OR “modifications” (All Fields)) AND (“heart disease risk factors” (MeSH Terms) OR (“heart” (All Fields) AND “disease” (All Fields) AND “risk” (All Fields) AND “factors” (All Fields)) OR “heart disease risk factors” (All Fields) OR (“cardiovascular” (All Fields) AND “risk” (All Fields) AND “factors” (All Fields)) OR “cardiovascular risk factors” (All Fields)) AND (“erectile dysfunction” (MeSH Terms) OR (“erectile” (All Fields) AND “dysfunction” (All Fields)) OR “erectile dysfunction” (All Fields)). There were no limitations in terms of publication date or study design.

## 3. Cardiovascular Risk Factors and Their Role in ED

A risk factor is defined as “an attribute or exposure that is associated with an increased probability of a specified outcome, such as the occurrence of a disease” [11]. Risk factors are traditionally divided into modifiable and non-modifiable. Modifiable risk factors are of particular interest because they can be improved through specific preventive interventions.

A true milestone in terms of the identification of risk factors for CVDs is the Framingham Heart Study (FHS). By popularizing the notion of “risk factor”, the FHS made a huge contribution to the development of preventive medicine, changing the focus from the treatment of patients with established CVDs to the prevention of the disease in people at higher risk.

The FHS is a longitudinal community-based study initiated in 1948 in the town of Framingham, located 30 km west of Boston. The initial cohort enrolled in the study consisted of 5209 inhabitants (about one-fifth of the entire city population) free of overt CVD. More than half of the sample was represented by women and the age range was 28–74 years [12]. The first 4 year follow up data were published in 1957 and described an association between both high cholesterol levels and hypertension and the development of IHD [13]. Over the following years, the FHS identified several other major important CV risk factors, focusing not only on IHD but also on other CVDs such as stroke and heart failure. During the second half of the 20th century, many other cohort studies have confirmed and expanded the notions derived from the FHS.

Since atherosclerosis is the underlying pathology of the great majority of overt CVDs, all the risk factors for the development of an atherosclerotic plaque should be considered relevant to CVD. Among non-modifiable CV risk factors, age, gender, family history for CVD and ethnicity should be mentioned.

Age is indeed a measure of exposure time, and it is positively related to CV risk representing the strongest predictor of adverse cardiovascular outcome [14,15]. Male gender is another well-established CV risk factor. Data from the FHS showed that CV mortality for women is comparable to that of men 10 years younger [16]. Another well-established non-modifiable risk factor is a positive family history for CV events in a first-degree relative [17,18]. This association is particularly strong in younger individuals with a positive family history of premature disease [19,20]. Although non-modifiable, the identification of these risk factors is of great clinical relevance because they help identify men who warrant stricter control of the modifiable CV risk factors. Among the latter, hypertension, dyslipidemia, diabetes mellitus (DM) and tobacco smoking are the most widely known and strongly related to MACE.

Hypertension is a well-established risk factor for all major atherosclerotic manifestations, including IHD, stroke and PAD [21,22]. There is a strong, direct and continuous relationship between blood pressure (BP) and CVD, without any clear evidence of a threshold for risk in the range of usual BP (>115/75 mmHg) [22]. This association is documented in all ages and it seems to be stronger for systolic than diastolic BP [23]. The relative risk is higher in adults of 40–69 years old, where an increase of 20 mmHg in systolic BP or 10 mmHg in diastolic BP is associated with more than a two-fold increase in stroke or IHD mortality [22].

Several lipid abnormalities are related to atherosclerotic disease. Both high total and low-density lipoprotein cholesterol (LDLc) are strongly associated with the development of IHD and, to a lesser extent, of stroke [24,25]. As with hypertension, this is a continuous relationship without a clear threshold [26]. Conversely, an increased risk for IHD is associated with low levels of high density lipoprotein cholesterol (HDLc) [27,28]. Data for the relationship between triglycerides and incident MACE are more controversial. Increased serum triglycerides are associated with three to five-fold higher risk of IHD and stroke and with doubled mortality in the general population [29,30] although high triglycerides in subjects with successful treatments for lowering LDLc were not associated with an increased risk in all studies [31] increased risk in all studies [31]. Another factor related to lipid is lipoprotein (a) (Lp(a)). This is a type of LDL in which apolipoprotein B-100 is covalently linked to a large glycoprotein named apolipoprotein A. High circulating Lp(a) is an independent risk factor for the development of CVD [32]. Apolipoprotein A is a highly polymorphic protein and 90% of the variability of plasma Lp(a) concentration is genetically determined [33]. Therefore, this may be considered a non-modifiable risk factor although phase 3 studies are ongoing for Lp(a) lowering medications (https://clinicaltrials.gov/ct2/show/NCT04023552 accessed on 12 May 2021).

Diabetes mellitus provides a two to three-fold increased risk of myocardial infarction or stroke and a two-fold increased risk of CV mortality [34]. This risk increases with the degree of glycemic alteration [35]. Borderline abnormalities of glucose metabolism (i.e., impaired glucose tolerance and impaired fasting glucose) have also been associated with an increased risk of CVD [36]. In addition to diabetes itself, diabetic patients also have an increased CV risk related to the frequent compresence of other metabolic risk factors.

Cigarette smoking is an important risk factor for CVD morbidity and mortality. It has been associated with a two to four-fold increased risk of IHD and with an elevated risk of sudden death [37]. The relative risk of myocardial infarction is proportional to tobacco consumption, higher in women than in men, and for inhalers compared with non-inhalers [38].

Additional established CV risk factors are obesity [39], chronic kidney disease [40], low socioeconomic status [15], low consumption of fruits and vegetables [41], low level of physical activity [42] and high levels of C-reactive protein [43].

Not surprisingly, many CV risk factors also increase the risk of developing ED [44]. Several studies have focused on the relationship between ED and traditional CV factors. Meta-analyses have found an increased risk of ED in hypertensive (odds ratio (OR) ranging 1.54 to 1.74) [45,46] or diabetic (OR ranging 2.08 to 3.62) [45,47] subjects. Hypertension favors endothelial dysfunction, which is the pathogenic link underlying the relationship between ED and CVD, by reducing endothelium-dependent relaxation and nitric oxide (NO) availability, and by increasing plasma endothelin concentrations [48], oxidative stress, and vascular hypertrophy [49]. In diabetes also, many pathological pathways may prompt endothelial dysfunction and vasoconstriction, including a reduced endothelial NO synthase (eNOS) activation caused by insulin-resistance [48] a greater depletion of the intracellular reduced form of Nicotinamide Adenine Dinucleotide Phosphate (NADPH) pool caused by hyperglycemia, an excessive endothelial and vascular smooth muscle proliferation due to an abundance of growth factors, and an increased activation of inflammatory pathways and oxidative stress [49]. Both high total cholesterol and low HDLc levels have been associated with an increased risk for ED that is respectively more than thrice higher and almost doubled for each mmol/L change in these lipid fractions [50]. Dyslipidemia also affects endothelial function through a faster inactivation of NO, most probably caused by increased L-arginine depletion, which, in turn, leads to high levels of intracellular reactive oxygen species (ROS) [48]. Obesity, and in particular visceral fat accumulation, is another independent risk factor with a 3% increased frequency of ED for each 1 cm increase in waist circumference [51]. Furthermore, a meta-analysis of the available population-based studies has shown that current smoking is associated with a 50% increased risk of ED and the risk is not nullified by quitting smoking as shown by the 27% increased risk found in past smokers [52]. For current cigarette smoking, a significant dose-response effect was also found with a higher number of cigarettes per day associated with a greater risk of ED [52]. Physical inactivity is another risk factor for ED as proven by a limited number of studies, which overall show a 20% increased risk in non-exercisers as compared with exercisers with greater benefit from moderate physical activity, but no further improvement from high-intensity exercise [52].

## 4. Calculators for CV Risk Estimation

The formulation of the concept of risk factors by the Framingham investigators and the quantification of different strengths for their association with the development of MACE underlined that, for each individual, overall risk is provided by the interaction of multiple factors that should be identified and considered as a whole. Accordingly, treatment strategies must be implemented taking into account the global risk rather than the presence of single conditions. This approach laid the groundwork for the development of multivariate risk models. The application of these models permits clinicians to estimate the individual risk for the first MACE based on the presence of different risk factors included in an algorithm. A variety of risk models and risk calculators have been developed over the years, most of them derived from large prospective studies [53,54,55,56,57,58,59,60,61,62]. Table 1 summarizes the characteristics of the main ones. Despite some differences, most of these include age, gender, blood pressure, total cholesterol, DM, and current cigarette smoking. Based on these, they provide the absolute risk (the probability of the adverse event happening) or, less frequently, the relative risk (comparison of the absolute risk of an individual with an age- and sex-matched subject without risk factors), as in the case of the SCORE risk algorithm [63]. According to the calculated probability of developing CVD, patients are classified as being at low-, intermediate- or high-level risk with a consequent array of therapeutic approaches ranging from lifestyle recommendations to aggressive primary intervention pharmacological treatment.

Indeed, these are simple tools that proved to be very useful for CV risk assessment and their use became increasingly widespread both in routine clinical practice and for modulating the healthcare system policies in several countries. However, they have some drawbacks that should be considered. Firstly, clinicians should be aware that the highest accuracy of these calculators is achieved when they are applied to populations with the same characteristics of the original validation cohort. The risk prediction is indeed considerably unreliable when used in specific subsets of patients such as ethnic minorities, very young or very old ages, patients with specific comorbidities, or even in populations with a different sociodemographic background. For instance, the Framingham risk function derived from US data proved to overestimate the absolute risk in European populations [54,64]. Thus, the specific characteristics of each patient need to be carefully taken into consideration when choosing a particular CV risk calculator.

Besides applicability, other weaknesses should be recognized. The majority of current risk calculators assess the risk of developing CVDs over a defined period of time (usually 10 years) without considering lifetime risk. Lifetime risk may be considerably higher in younger patients and lead to more aggressive risk factor reduction strategies. Age is the strongest determinant of risk in most CV calculators. Thus, a young person is very likely to be classified as at low-risk, even in the presence of multiple major risk factors. For instance, according to the SCORE algorithm, a 40-year-old man is not placed in the high risk-category even if severely hypertensive, severely hypercholesterolemic and a current smoker. A similar feature occurs using the Framingham function [65]. This also introduces the issue of absolute risk, which may be an ineffective manner in which to communicate the need for a correct adherence to medical therapy or for the modification of lifestyle in younger subjects.

The restricted number of major risk factors considered by the CV risk calculators represents a further limit. This simplifies the use of the charts and provides a high specificity because patients identified as at high risk surely need intensive primary prevention interventions. On the other hand, it has a relatively low sensitivity as it may lead to an underestimation of CV risk in patients with multiple borderline abnormalities or with a series of minor risk factors not included in the model [66].

Some of these limitations have been partially overcome by the Joint British Societies 3 (JBS3) Board risk calculator [59] that presents some unique and interesting features. First, the JBS3 calculator permits an estimation of lifetime CV risk, which is less dependent on age and gender. This is a better approach when dealing with patients such as young individuals with multiple modifiable risk factors that result in low short-term risk but in high lifetime risk. Furthermore, it offers some additional metrics such as “vascular age” (see below) and expected CVD event-free survival age, along with different graphics showing how different lifestyle modifications and other interventions would reduce the risk of a CV event. These novel tools could represent an effective way to improve CV risk communication and to explain the importance of specific interventions by the clinicians. Validation in populations other than British would be important for a larger applicability.

Although current algorithms for CV risk estimation include the strongest risk factors, a great number of CV events still occur in people classified as low- or intermediate-risk. The difference between the estimated and observed CV events, or “residual risk”, may be explained by the presence of factors not included in standard risk models. These additional factors, both environmental and genetic, may play an important role in the determination of CV events in patients misclassified as at low-risk.

Many professional societies have considered the evaluation of additional risk factors, such as the 2018 American professional society guideline on cholesterol management that introduced the expression of “risk-enhancing” factors [67]. Important efforts are being made to identify other markers that can limit the residual risk through the reclassification of individuals in higher-risk categories. The evaluation of additional risk factors may be particularly useful in specific categories of patients: (i) younger individuals, easily misclassified as low-risk patients, (ii) subjects without traditional risk factors but with several secondary predisposing conditions not included in current risk calculators, and (iii) patients whose estimated risk calculated by standard CV risk engines falls near a threshold for treatment. In these cases, considering additional risk factors may considerably alter the risk estimation and guide primary prevention treatment decisions.

## 5. Beyond the Classical Risk Factors: Identifying ED Men at Higher CV Risk

**Figure 1:**
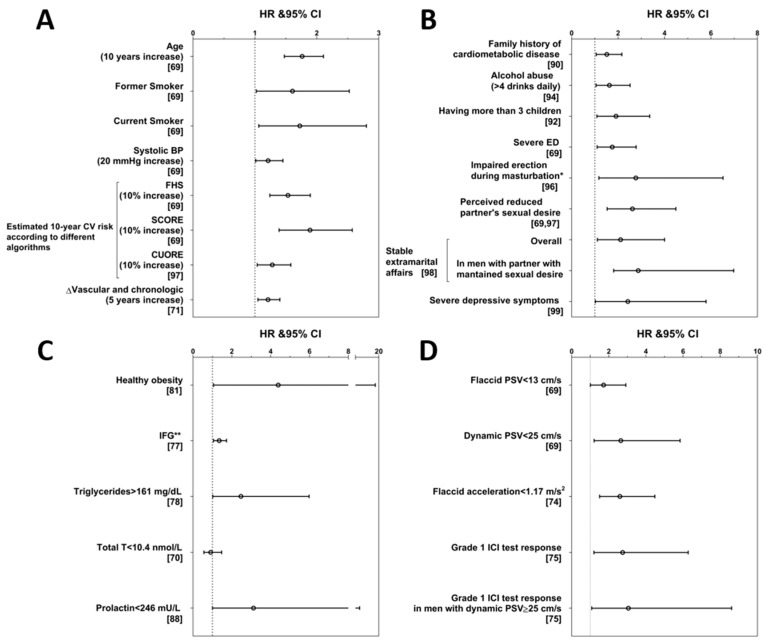
Conventional and unconventional risk factors associated with major adverse cardiovascular events in subjects with erectile dysfunction. Risk of major adverse cardiovascular events (MACE) over 4.3 years of median follow-up in a population of 1687 men consulting the Andrology Unit at the Careggi Hospital, University of Florence (Florence, Italy) for sexual dysfunction, according to conventional and unconventional risk factors as measured at baseline. (**A**) risk of MACE associated with conventional risk factors; (**B**) risk of MACE associated with unconventional risk factors derived from personal and sexual history; (**C**) risk of MACE associated with unconventional risk factors derived from metabolic factors and hormones; (**D**) risk of MACE associated with unconventional risk factors derived from instrumental tests. Data are expressed as hazard ratio (HR) and 95% confidence interval (CI) and are derived or adapted from original publications. The original publication is quoted in brackets for each parameter. * Limited to non-diabetic patients without severe ED; ** The outcome is cerebrovascular events. BP = Blood Pressure; CV = cardiovascular; ED = Erectile Dysfunction; FHS = Framingham Heart Study; ICI = intra-cavernous injection; IFG = Impaired Fasting Glycemia; PSV = peak systolic velocity; SCORE = Systematic COronary Risk Evaluation; T = Testosterone.

In ED men, a population enriched with CV hazard, a meticulous evaluation of risk factors and the use of multiparameter algorithms for quantifying risk may still leave out subjects at high risk due to CV risk factors not captured by traditional risk factor assessment. This point has been taken on board by the last version of the QRISK calculator (QRISK3, Table 1), which incorporated ED diagnosis or treatment in men as a further variable for CV risk estimation [58]. The extent of the risk denoted by having ED is well described in the Olmsted County study, a population based survey on more than 1400 men aged 40 to 79 years [68]. This has shown that, among men aged 40–49 years, and therefore at considerably low CV risk, the incidence of IHD in ED men was almost 50-fold higher than in men without ED [68]. With ageing, the power of ED as a CV risk marker was greatly attenuated, so much so that in men aged 70–79 years with or without ED the incidence of IHD is largely the same [68]. It is therefore pivotal to identify markers that in ED men could fill in the gap for their residual risk. Over the last decade, our group has been deeply committed to evaluating the possible factors that could be useful predictors of MACE in ED subjects. We studied this topic in a consecutive series of 1687 men consulting our Andrology Unit at the Careggi Hospital, University of Florence (Florence, Italy) for sexual dysfunction. For these subjects, we retrospectively collected data on incident MACE through administrative registries of hospital discharge using the definitions of the International Classification of Disease. The methods and definitions have been previously extensively reported [69,70]. During a median follow-up of 4.3 years, 139 MACE occurred; 85 were IHD, 40 were cerebrovascular events, and 14 were PAD. Fifteen MACE were fatal. Several traditional risk factors were associated with the new occurrence of MACE, including age, systolic BP, current and former smoking habits, and DM [69] (Figure 1A). Not surprisingly, subjects with higher estimated CV risk according to the Framingham, the SCORE (Systematic COronary Risk Evaluation) and the Progetto Cuore algorithms, respectively derived from the US, European and Italian populations, had an increased incidence of MACE [69,71] (Figure 1A). As aforementioned, age bears a great weight in these algorithms and, in the attempt to unveil subjects at higher risk among those with apparently lower predisposition, the construct of vascular age was applied to our population. This concept has been introduced in order to easily explain the high relative CV risk in younger subjects that have low absolute risk. In a man with a combination of modifiable risk factors, this corresponds to the chronological age of a man with the same estimated CV risk but without any modifiable risk factor. The calculation of the difference between vascular and chronological age, according to the SCORE algorithm, denoted that a higher difference was associated with higher incidence of MACE, even after adjustment for confounding factors [71] (Figure 1A). Interestingly, in subjects younger than 57 years, those with more than 9 years of difference were at the highest risk [71]. Conversely, in older men as well as those without metabolic derangements or previous CVD, this parameter did not provide more insight than traditional risk factors [71].

### 5.1. Unconventional CV Risk Factors: Parameters Derived from Instrumental Assessment

A great deal of information could be obtained by the tests that are commonly used in the diagnostic workup of ED. The assessment of penile vascular efficiency is the most obvious starting point to unearth new possible markers of CV risk. These tests represent valuable tools for ED diagnosis. As detailed below, they offer important information on CV risk and their use is therefore greatly encouraged in specialized (endocrinology, andrology, urology) settings.

Decreased peak systolic velocity (PSV) of the penile artery blood flow after the intra-cavernosal injection of prostaglandin E_1_ (PGE_1_, dynamic evaluation) at baseline was found as a significant predictor of incident MACE over the follow-up period [69]. After adjustment for confounders, dynamic PSV < 25 cm/s, which is a recognized threshold for the definition of arterio-genic ED [72], maintained its association with a more than two-fold increased risk of MACE [70] (Figure 1, Panel D). Despite dynamic penile color Doppler ultrasound (PCDU) evaluation being a standard of care for ED patients, this is not devoid of risks, besides being more expensive. Therefore, we evaluated the reliability of flaccid PCDU parameters in predicting MACE. Flaccid PSV < 13 cm/s has been previously reported as a marker of asymptomatic IHD in diabetic patients [73]. When baseline flaccid PSV < 13 cm/s was checked as a possible predictor, we found that it was associated with an almost doubled risk of incident MACE [69] (Figure 1D). Flaccid acceleration is another useful PCDU parameter because values below 1.17 m/s^2^ not only are independent predictors of incident MACE (Figure 1D), but they retain their significant association with forthcoming MACE in patients commonly considered at low risk, such as younger, non-obese, non-diabetic and normotensive subjects [74]. Interestingly, the effect of flaccid acceleration as a CV risk marker is independent of dynamic PSV values, [74] thus indicating that this captures different information for CV risk stratification. PCDU is not a universally available instrument for the assessment of ED and is often replaced by an easier and less expensive tool that is the intra-cavernous injection (ICI) test. This consists in evaluating on a four-point scale the extent of an erection after the injection in the corpora cavernosa of a vasodilating agent, such as PGE_1,_ papaverine or phentolamine. The grade 1 response corresponds to a lack of erection and the grade 4 to a complete erection. In our population, a grade 1 response at baseline was associated with an adjusted hazard ratio (HR) for forthcoming MACE of 2.75 (1.20–6.28) vs. the higher grade of response [75] (Figure 1D), thus comparable to what is associated with low dynamic PSV, flaccid PSV or acceleration [69,74]. Interestingly, grade 1 response at ICI test had an even stronger association with MACE occurrence (HR = 3.05 (1.08–8.62)) in subjects with normal dynamic PSV [75] (Figure 1D) thus providing further and complementary information on CV risk.

### 5.2. Unconventional CV Risk Factors: Metabolic and Hormone Parameters

According to the Princeton III recommendations for the management of ED [76], glycolipid parameters should be measured for the assessment of still undiagnosed DM or dyslipidemia. The wide availability of most of these measurements represents a great opportunity to perform CV risk stratification of ED men in non-andrological settings (GP, internal medicine). Therefore, it is important that general practitioners be sensitized to check for these values and interpret the results in light of the high CV risk of ED men.

Diabetes mellitus and total or LDL cholesterol are traditional risk factors that are included in most algorithms. However, further information may be collected by assessing the glycolipid profile. When considering subjects with ED and impaired fasting glucose, we showed that they risk experiencing a MACE, and in particular a cerebrovascular event, higher than subjects with normal fasting glucose but lower than diabetic men [77] (Figure 1C). This shows that, although glycaemia is often interpreted as a dichotomous variable where diabetes is in contraposition with normality, impaired fasting glucose is not devoid of CV risk. In the same population of ED men, baseline triglyceride levels >161 mg/dL (corresponding to lower limit of the highest quartile) were associated with an increased risk of MACE with an adjusted HR of 2.47 (1.02–5.98) [78] (Figure 1C). Lipoprotein (a) may represent a further potential unconventional risk factor. Its measurement is not recommended for routine clinical practice and, therefore, it is not included in most algorithms. However, adding Lp(a) to traditional risk factors in prediction models has been demonstrated to improve estimation [79]. No studies have so far evaluated the role of Lp(a) in predicting MACE in ED subjects. Since these metabolic derangements are often complications of obesity, it is to be expected that metabolic syndrome is associated with increased CV risk [80]. However, the role of non-complicated obesity—the so-called “healthy obesity”—as a CV risk factor is less obvious. In our population of ED men, those that had an increased body mass index ≥30 kg/m^2^ with normal HDLc, normal BP and non-diabetic had a higher incidence of MACE than non-obese men and a similar rate to obese men with complications [81] (Figure 1C).

Among biochemical markers, hormone levels have also been tested as possible predictors of MACE. The role of Testosterone (T) in CV health is controversial. In our population of ED men, low T was not a significant predictor of MACE [70] (Figure 1C) and it was even protective when only obese subjects were selected [82]. However, serum T below 10.4 nmol/L was associated with a six-fold increase in MACE lethality [70]. These data are consistent with previous meta-analyses [83,84] and overall suggest that low T, particularly in unhealthy men, is a marker of frailty more than being directly involved in CV health. However, it should be recognized that the most recent meta-analysis on this topic has shown that hypogonadism is associated with an increased incidence of MACE [85], thus questioning the lack of specific effects on CV risk.

Prolactin is a hormone whose physiologic function in males is still largely unknown. Its biological activity becomes evident when it rises to overt pathological levels [86]. However, the effects of prolactin in the lower range of values have scarcely been investigated. We pioneered the study of this topic in subjects seeking medical care for sexual dysfunction. Besides showing that lower prolactin is associated with more severe anxiety symptoms, premature ejaculation and an adverse metabolic profile [87], we reported for the first time that low prolactin at baseline (I–IV quintile) is associated with forthcoming MACE, after adjustment for possible confounders (Figure 1C), and a 6% increase in risk for each 10 ng/mL of reduction in baseline prolactin was found [88]. Nevertheless, this correlation was apparently not a direct effect of prolactin [87,88] and we hypothesized that low prolactin could be a circulating marker of low brain serotonergic tone. Although this remains a speculation, subsequent epidemiological studies in different clinical settings and in general populations have confirmed our results [89].

### 5.3. Unconventional CV Risk Factors: Personal History

Most of the aforementioned markers pertain to evaluations and tests that are commonly prescribed and interpreted in specialized settings. However, results from studies on our cohort also suggest that a thorough assessment of personal history and ED characteristics using simple questions may also help in CV risk stratification. Among the most sensitive topics, we recently confirmed that having a family history of CVD predisposes patients to MACE [90]. Besides confirming this known—though largely neglected—risk factor, we showed that family history for DM is also a risk factor for MACE [90] (Figure 1B). Moreover, family history for cardiometabolic disease proved to be a significant risk factor particularly in subjects without further conventional risk factors such as in younger (<55 years old), non-diabetic, non-dyslipidemic men, without previous CV events or other chronic diseases, with HRs ranging 1.63 to 2.28 [90]. Still concerning personal history, according to previous evidence [91], we found that fatherhood is associated with worse metabolic profile and is a predictor of forthcoming MACE with a dose dependent effect that is highest for men having more than three children [92] (Figure 1B).

Smoking habits were expectedly confirmed as risk factor in our ED population [69]; however, the finding of a significant association regarding the relationship of alcohol intake and incident MACE is less obvious. In fact, alcohol drinking has been variously associated with CV risk with data showing either negative, positive or U-shaped association with number of drinks and incident MACE [93]. In our sample, subjects having more than four drinks per day had an almost two-fold increased risk of experiencing a MACE over the following 4.3 years of follow-up [94] (Figure 1B).

### 5.4. Unconventional CV Risk Factors: Characteristics of ED and Sexual Life

Thoroughly investigating ED could provide useful background for clinical management but also for CV risk stratification. These data could be obtained through a few simple questions that can be asked in both specialized (endocrinologists, andrologists or urologists) and non-specialized (GPs, specialists in internal medicine or in cardiology) settings spending a small amount of time in order to gain a great deal of information.

Self-reported ED, if adequately graded, is a valuable tool for risk prediction. Severe ED, self-defined as difficulties in achieving an erection sufficient for penetration in >75% of sexual attempts, was associated with a 75% increased risk of forthcoming MACE [69] (Figure 1B). Not only the quality of erection but also the frequency of sexual intercourse provides information on CV health. In particular, lower frequency of sexual intercourse was associated with significantly increased risk of forthcoming MACE. Even more interestingly, this was confirmed after excluding men with severe ED, thus showing that investigating the frequency of sexual intercourse could give additional information when compared to the simple assessment of ED [95]. Similarly, asking about the quality of erection during autoeroticism is important for qualifying ED in men not having regular intercourse, in whom the assessment of sex-related erection may not be reliable. Men reporting impaired erection during masturbation had a three-fold increased incidence of MACE (Figure 1B), even when the analysis was restricted to men without severe ED and to those classified as at low risk according to age (younger than 55 years old) or glycaemia (non-diabetic) [96].

It should always be borne in mind that sexual problems are dyadic and partner’s sexual problems should always be assessed when approaching men consulting for ED. In our clinical practice, we systematically evaluate several partner’s problems as perceived by the patient. Among these, we quite surprisingly found that a patient that perceives a reduced sexual interest in his partner has worse CV outcomes [69] (Figure 1B). It is interesting to note that the perception of decreased sexual interest in the partner is, together with a lower education degree, the parameter that most reliably predicts MACE in our population of ED men [97]. However, when men cheat on their partners, the problem reverses and men with extramarital affairs that perceive good sexual desire in their primary partner are the most at risk of experiencing new MACE [98] (Figure 1B).

Finally, in our sample, besides relational and organic issues evaluated as pathogenic components of ED, we also assessed psychological symptoms and confirmed the role of depressive symptoms as predictors of forthcoming MACE [99] (Figure 1B), in line with data from different settings.

## 6. From CV Risk Identification to CV Risk Modification

From the previous paragraphs, it is evident that ED patients are subjects at high CV risk. ED is an early manifestation of systemic arterial disease [8,100,101] and ED populations suffer from many other traditional risk factors [102,103]. The other side of the story is that ED provides the unique chance to investigate CV health and risk factors and, through the careful assessment of the erectile problem, to identify non-traditional predictors that may refine the CV risk estimation. The recognition of men at higher risk could lead to undertaking earlier measures to improve metabolic and CV health, so that, in this view, ED subjects could be considered “lucky” [104]. Taking care of CV health in ED subjects is indeed a great goal for clinicians. However, men seeking medical care for ED are interested in improving their sexuality. Hence, are the strategies for the improvement of risk factors effective also in improving erectile function?

Several lines of evidence have shown that physical activity has a positive impact on erectile function, in particular with moderate and intense physical activity associated with a lower risk of ED [105]. A recent systematic review [106] focused on the levels of physical exercise needed to improve ED and found that 40 min of aerobic, moderate intensity physical activity, 4 times a week for 6 months improves erectile function in men with ED. There is evidence that the advantages regarding erectile function associated with physical activity are due to its beneficial effects on endothelial function. In particular, regular physical exercise has the potential to empower eNOS synthesis and activity [107,108] as well as to increase circulating regenerative endothelial progenitor cells [109].

In the 2000s, Esposito et al. examined the impact of diet on ED, revealing that the Mediterranean diet is effective in improving ED in obese patients with metabolic derangements [110,111]. Later, the beneficial effects of this dietary regimen on erectile function were shown also in diabetic patients, with a lower incidence of ED over more than 8 years of follow-up among men following a Mediterranean diet with closer adherence when compared with the less compliant groups [112]. The positive effects of dietary modifications may be explained by the beneficial impact of weight loss on endothelial function, insulin sensitivity, and circulating T levels that eventually lead to improved erection [113,114,115]. A randomized clinical trial (RCT) on 100 obese men with ED showed that an intensive weight loss program (diet and physical exercise) caused positive changes in physiologic measures of endothelial dysfunction and improved erectile function [116]. A following study performed on diabetic men [117] showed that diet and physical activity were able to preserve the erectile function, although they were not effective in actually improving it. This could be explained by underlying neurogenic and arterio-genic dysregulations that are common in diabetic patients and that could limit the reversibility of ED.

In recent years, bariatric surgery has increased as an important tool for weight loss in severe obesity. Bariatric surgery has shown a positive impact on ED [118,119] that may be explained by weight loss [120], the increase in total and free T [115], the sudden decrease of glycaemia [121] or the general amelioration of DM-related metabolic dysfunction, as demonstrated in both animal models [122] and humans [118]. Other authors have suggested that ED improvement may be independent of BMI and weight loss and could be related to changes in endothelial function [123].

Smoking cessation has been associated with a partial improvement of penile artery flow both in animal models [8] and in humans [124,125]. An RCT, published in 2010, showed that smoking cessation was associated with a two-fold higher probability of ED improvement, as compared with non-quitters, after a follow-up of 6 months [126]. These results are consistent with a following study that observed a significant improvement of erectile function after 8 weeks of follow-up in the successful quitters group, compared with the unsuccessful [127].

Poor glycemic control in diabetes is associated with severe ED [128], but few studies have examined the relationship between glycemic control and ED severity. A 2012 Korean study on streptozotocin-induced diabetic rats showed that glycemic control improves several makers of erectile function, but only the strictest control can lead to almost complete recovery from this condition [129]. Aside from glycemic control, antidiabetic drugs may have a role in ED improvement. Metformin, an antidiabetic drug discovered sixty years ago, reduces body weight [130,131], inflammatory response [132], oxidative stress and ameliorates ED in human and animal studies [133]. A putative mechanism through which metformin could play a beneficial effect on erectile function is the improvement in cavernosal NO signaling [133]. According to a rabbit model of metabolic syndrome induced by a high fat diet, also characterized by hyperglycemia, the treatment in vivo with metformin is able to stimulate the adenosine-induced smooth muscle cell relaxation in penile corpora cavernosa [134]. Data on humans are scanty. Only one RCT on a small sample has been performed so far showing that metformin associated with sildenafil improves ED significantly more than sildenafil+placebo [135].

Our knowledge of the role of novel antidiabetic agents is still limited. Sodium-Glucose Transport Protein 2 (SGLT2) and Glucagon-Like Peptide-1 Receptor Agonists’ (GLP1RA) effect on weight loss could improve gonadal and sexual function in diabetic patients [136]. In a rabbit model of streptozotocin-induced diabetes, GLP1RAs, administered both in vivo and in vitro, appear to blunt smooth muscle dysfunction by downregulating the Ras homolog gene family and Rho-associated protein kinase 2 pathway and to reduce ROS production, independently of glucose or weight-lowering effects [137]. In men, only one small observational trial using liraglutide in obese diabetic men is available, although showing encouraging results [138]. Knowledge about the effect of SGLT2-inhibitors and Dipeptidyl Peptidase-4 (DPP-4) inhibitors are even more limited. Preclinical data on the former suggest an improvement in NO synthesis and release in cavernous tissue [139]. DPP-4 inhibitors have proved to improve endothelial function and reduce atherogenesis in several arterial districts [140,141,142].

In a rat model of hyperlipidemia and ED, dietary changes were linked to positive effects on penile hemodynamics [143]. Chronic statin treatment, through its anti-oxidative effects on the endothelium, seems effective in restoring erectile function in diabetic rats [144,145].

## 7. Conclusions

Erectile dysfunction is an early manifestation of cardiovascular disease. For this reason, ED should be considered an alarm bell. Men with ED should be carefully assessed for CV risk factors in order to prevent future MACE. It is, however, known that traditional risk factors do not completely catch all subjects at high CV risk. In fact, a relevant proportion of MACE occurs in men apparently free from risk factors. In men with ED, who should be considered at high CV risk until proven otherwise, it is important to take into account not only traditional risk factors but also unconventional ones. Several parameters that derive from good clinical practice assessment of subjects with ED proved to be valuable predictors of MACE. Among these, the assessment of family history of cardiometabolic events, alcohol abuse, fatherhood, partner’s sexual interest and the severity of the impairment in erection during intercourse or during masturbation should be mentioned. In addition, alteration in metabolic parameters demonstrated their predictive value, including impaired fasting glucose, slightly increased triglycerides, and obesity without metabolic complications. Decreased penile blood flow upon intra-cavernosal injection of vasodilating agents has been associated with increased CV risk. However, data from other simpler and less expensive instrumental tests have similar strength as MACE predictors, including peak systolic velocity and acceleration of penile blood flow measured in flaccid conditions and the response to an intra-cavernosal injection test that does not require Doppler ultrasound. Recognizing these risk factors may help in identifying, among subjects with ED, those that warrant stricter lifestyle or pharmacological interventions to minimize their CV risk. Taking care of a patient with ED is an opportunity to implement a strict correction of modifiable risk factors. Furthermore, this may be not only a strategy to decrease CV risk but also a way to improve sexual complaints.

## Figures and Tables

**Table 1:** Characteristics of the main algorithms for cardiovascular risk estimation.

Characteristics	Framingham Risk Score 2008 [53]	SCORE [54]	PROCAM [55]	QRISK1 [56], 2 [57], and 3 [58]	JBS3 Risk Calculator [59]	Reynolds [60,61]	CUORE [62]
Cohort type	General population; US	Mainly general population, some occupational cohorts; EU	Healthy employees; Germany	Healthy general practice attendees; England/Wales	Healthy general practice attendees; England/Wales	Health professionals; US	General population; Italy
Cohort size (% men)	8491 (47%)	205,178 (57%)	26,975 (68%)	QRISK1: 1.28 million (50%) QRISK2: 2.29 million (50%) QRISK3: 2.92 million (50%)	2.29 million (50%)	35,332 (40%)	20,647 (37%)
Age range (years)	30–75	40–65	20–75	QRISK1 and 2: 35–74 QRISK3: 25–84	35–74	45–80	35–69
Endpoint assessed	10-yr risk of CVD events (CHD death, myocardial infarction, coronary insufficiency or angina, coronary revascularization, stroke/TIA, PAD, heart failure)	10-yr risk of CVD mortality (including CHD, arrhythmia, heart failure, stroke, aortic aneurysm, and PAD)	Two different scores: 10-yr risk of CHD 10-yr risk of stroke	10-yr risk of CVD events (CHD death, myocardial infarction, coronary insufficiency or angina, coronary revascularization, stroke/TIA, PAD)	10-yr and lifetime risk of CVD events (CHD death, myocardial infarction, coronary insufficiency or angina, coronary revascularization, stroke/TIA, PAD); Effect of risk factor optimization; Heart age; CVD-free life-expectancy	10-yr risk of CVD events/mortality (myocardial infarction, stroke, coronary revascularization)	10-yr risk of CVD events (myocardial infarction, stroke)
Variables included	Sex, age, total cholesterol (mg/dL), HDL cholesterol (mg/dL), SBP (mmHg), current smoking (yes or no), diabetes (yes or no), hypertensive treatment (yes or no)	Sex, age, total cholesterol (mg/dL) or total cholesterol/ HDL cholesterol ratio, SBP (mmHg), current smoking (yes or no) High- and low-risk countries version Most diabetic patients considered high-risk regardless of other factor	Sex, age, LDL cholesterol (mg/dL), HDL cholesterol (mg/dL), SBP (mmHg), current smoking (yes or no), diabetes (yes or no),	QRISK1: Sex, age, total cholesterol/ HDL cholesterol ratio, SBP (mmHg), current smoking (yes or no), hypertensive treatment (yes or no), area-based index of poverty, family history of premature CVD in first degree relative (yes or no), BMI (kg/m^2^) QRISK2: also includes ethnicity, diabetes (yes or no), rheumatoid arthritis, chronic renal disease, and atrial fibrillation QRISK3: migraine, medications (corticosteroids and atypical antipsychotics), systemic lupus erythematosus, severe mental illness, SBP variability, erectile dysfunction	Sex, age, total cholesterol/ HDL cholesterol ratio, SBP (mmHg), current smoking (yes or no), hypertensive treatment (yes or no), area-based index of poverty, family history of premature CVD in first degree relative (yes or no), BMI (kg/m^2^), ethnicity, diabetes (yes or no), rheumatoid arthritis, chronic renal disease, and atrial fibrillation	Sex, age, total cholesterol (mg/dL), HDL cholesterol (mg/dL), SBP (mmHg), current smoking (yes or no), hs-CRP (mg/L), HbA1C if diabetic (percent), parental history of premature CVD (yes or no)	Sex, age, total cholesterol (mg/dL), HDL cholesterol (mg/dL), SBP (mmHg), current smoking (yes or no), diabetes (yes or no)
Important variables excluded	Family history of, CVD, BMI	Diabetes, Family history of CVD, BMI, Hypertensive treatment	Family history of CVD, BMI, Hypertensive treatment	None	None	BMI, Hypertensive treatment	Family history of CVD, BMI, Hypertensive treatment

SCORE = Systematic COronary Risk Evaluation; PROCAM = Prospective Cardiovascular Münster; JBS = Joint British Societies; BMI = Body Mass Index; CHD = Coronary Heart Disease; CVD = Cardiovascular Disease; EU = European Union; HbA1C = Hemoglobin A1c; HDL = High-Density Lipoproteins; hs-CRP = high-sensitivity C-Reactive Protein; LDL = Low-Density Lipoprotein; PAD = Peripheral Artery Disease; SBP = Systolic Blood Pressure; TIA = Transient Ischemic Attack; US = United States.

## Abbreviations

BP: blood pressure, FHS: Framingham Heart Study, SCORE: Systematic COronary Risk Evaluation, CUORE: Progetto Cuore, ED: erectile dysfunction, IFG: impaired fasting glucose, T: testosterone, PSV: peak systolic velocity, ICI: intra-cavernosal injection.
